# A Single-Centre Clinical Audit of Erectile Dysfunction Assessment and Management in UK General Practice Against National Guidelines

**DOI:** 10.7759/cureus.108311

**Published:** 2026-05-05

**Authors:** Adithya Ajith

**Affiliations:** 1 Internal Medicine, The University of Sheffield, Sheffield, GBR

**Keywords:** clinical picture of erectile dysfunction, diabetes mellitus-induced erectile dysfunction, erectile dysfunction, risk factors of erectile dysfunction, treatment for erectile dysfunction

## Abstract

Erectile dysfunction (ED) is a prevalent condition with significant psychosocial and cardiometabolic implications, particularly in ageing populations. This quality improvement project evaluated whether the assessment and management of ED at a community general practice aligned with contemporary clinical guidelines, including those from the British Society for Sexual Medicine and the European Association of Urology. A retrospective audit was conducted using electronic patient records from a cohort of individuals prescribed ED medications between July 2023 and July 2024. Fifty patients were randomly selected, excluding those diagnosed prior to 2017, to ensure relevance to current standards.

Three key criteria were assessed: appropriate biochemical investigation for underlying causes, documentation of cardiovascular (CVD) risk assessment, and prescribing of cost-effective first-line pharmacotherapy. Audit performance demonstrated that 41/50 patients (82%) received recommended blood tests (standard: 85%), 38/50 patients (76%) underwent documented CVD risk assessment (standard: 80%), and 45/50 patients (90%) were prescribed low-cost phosphodiesterase type 5 inhibitors (standard: 90%). While prescribing practices met expected standards, gaps were identified in comprehensive investigation and cardiovascular evaluation.

Findings suggest that although ED management at the practice is broadly aligned with national guidance, improvements are needed in systematic risk assessment and documentation. The observed pattern between incomplete investigations and lack of CVD assessment highlights the need for structured consultation templates and increased clinician awareness. Recommendations include the implementation of validated assessment tools, enhanced coding practices, and patient education on the relationship between ED and cardiovascular health. A re-audit is advised to assess the impact of these interventions. This project underscores the importance of holistic ED management in primary care, with implications for both patient outcomes and healthcare sustainability.

## Introduction

Erectile dysfunction (ED) is defined as the consistent inability to achieve or maintain a penile erection that is satisfactory for sexual performance [1]. The prevalence of this health issue increases with age and is predicted to affect 322 million men by 2025 [2]. Due to the psychosocial impacts of ED and known associations with cardiometabolic disease, it is an area of significant concern within primary care. The development of safe and effective treatments, along with improved understanding of its aetiology, has led to more patients seeking help for ED.

Societal taboos and outdated perspectives contribute to many men failing to engage with primary care adequately. Therefore, a formal assessment of ED must occur in this vulnerable population for early diagnosis and management of cardiovascular disease (CVD) [1]. The latest Care Quality Commission (CQC) inspection for Elmwood Family Doctors reports a higher-than-average percentage of people in the 75+ year group [3]. The report also states that 61% of registered patients are diagnosed with a chronic condition, compared to 54% nationally. These demographics emphasise the importance of ED management at this practice, factoring in age and chronic multimorbidity.

The diagnosis and treatment of ED patients must be comprehensive and in accordance with current best evidence. Hence, general practitioners (GPs) should consider recommendations outlined by relevant organisations such as the British Society for Sexual Medicine (BSSM) and the European Association of Urology (EAU). This audit specifically evaluated three key aspects of ED management: whether patients received appropriate biochemical investigations for underlying causes, whether cardiovascular risk assessment was documented, and whether cost-effective first-line pharmacological treatment was prescribed in accordance with national guidelines. The aim of this quality improvement project is to determine whether the assessment and management of ED in this general practice meet the agreed standards of care as published in recent guidelines.

## Materials and methods

Methodology

This audit was conducted at Elmwood Family Doctors, a community general practice that covers a population of 15,000 patients. A project of this nature on ED assessment and management at the practice was not performed before, making this an initial audit. All information regarding diagnoses, investigations, and prescriptions was collected from patient records on the SystmOne database. For the purpose of the audit, this work was registered as a service evaluation and formal ethical approval was waived. Since the active management of ED is a key focus of this project, patients were selected from a register of those who had been issued ED medications within the past 12 months, from July 2023 to July 2024. Read codes that were applied to highlight patients included “Erectile dysfunction”, “Impotence”, “Cannot get an erection”, and “Cannot sustain an erection”.

Overall, 281 patients were identified in the system, and for this audit, 50 were selected using a random number generator. In terms of exclusion criteria, it was determined that implementing a cut-off date would be appropriate, according to the most recent update on guidelines. Recommendations for ED by the National Institute for Health and Care Excellence (NICE) were reviewed in 2017, in conjunction with the BSSM publication on the same topic that year [1]. Consequently, any patient who had presented with their first episode of ED prior to 2017 was excluded, so that the quality of care could be fairly evaluated.

Current practice was assessed by searching through individual patient records, with particular attention to ED diagnosis and management. The "Repeat Templates" section illustrated the patient’s regular medications and was used to ascertain what ED drug was prescribed. The patient’s consultation history could be found in "Tabbed Journal", and was used to confirm when they attended their first ED appointment. This initial meeting, subsequent follow-ups, and investigations ordered around the time period were analysed to check if cardiometabolic risks were assessed.

Criteria (with standards in brackets)

Standards for this quality improvement project were developed following discussions with the GPs, nursing staff, and pharmacists at Elmwood Family Doctors. Important points were raised surrounding the underlying causes of ED and the proportion of patients with chronic diseases at the practice. Considering these, the standards for cardiovascular risk assessment and investigations were locally set at 80% and 85%, respectively. The standard for CVD assessment was set slightly lower in view of the time pressures faced by GPs locally and nationwide, causing difficulties in the completion of a comprehensive evaluation during a single appointment. Barriers to achieving targets may include the patient opting out of blood tests and unwillingness to participate in aspects of history taking, such as cardiac stratification. As for phosphodiesterase 5 inhibitors (PDE5Is), the standard for 90% was determined from OpenPrescribing statistics - the national median for the percentage of prescribed ED drugs other than generic sildenafil or tadalafil ranges from 5% to 10% [4].

*Has the Patient Been Assessed for Underlying Causes of Erectile Dysfunction Using Appropriate Blood Tests Such as Serum Haemoglobin A1c*,* Serum Lipid Profile, and Fasting Serum Total Testosterone? (85%)*

ED is often a symptom of another health-related factor or condition, and may be linked to personal stressors. Various components of the metabolic syndrome have been implicated, including triglyceride, high-density lipoprotein, and fasting blood glucose levels. Evidence has shown that diabetic patients demonstrate a three-fold increase in the risk of developing ED, and this is further exacerbated in those who have received an earlier diagnosis of diabetes [5]. This relationship can be explained by the microvascular complications of persistent hyperglycaemia, such as peripheral and autonomic neuropathy. For example, oxidative stress seen in diabetes can impair the function of the cavernous nerve, bulbocavernosus, and ischiocavernosus muscles, all of which are crucial for penile erection [6].

Testosterone is a major regulator of erectile function, and as a result, ED is one of the first presentations of hypogonadism. Additionally, low serum testosterone levels may be connected with metabolic syndrome - aromatase, present in the adipose tissue of obese men, is responsible for the conversion of testosterone to oestradiol, a notable cause of hypogonadism [5]. Although one abnormal test result is not sufficient to diagnose hypogonadism, NICE recommends arranging a repeat, as well as follicle-stimulating hormone (FSH), luteinizing hormone (LH), sex hormone binding globulin, and prolactin levels. These should be used alongside a focused history and examination for men with low libido, small testes, and absent male secondary sex characteristics.

Is There Evidence That a Cardiovascular Risk Assessment Had Been Performed, Based on Existing Risk Factors and Comorbidities? (80%)

ED is an accepted predictor of CVD because of shared risk factors, including older age, smoking, obesity, and hypertension. Men who present with ED may have two to five years before they develop coronary artery disease [5]. During this potential window, GPs have the opportunity to provide valuable discussion and formulate holistic treatment plans for these patients. The enhanced understanding of the link between ED and cardiovascular health can influence lifestyle modifications such as regular exercise, weight loss, and smoking cessation.

Guidelines from the EAU have described stratification of ED patients into low, medium, and high-risk categories, depending on the degree of hypertension, history of angina or myocardial infarction, and enquiry into exercise capacity [7]. In high-risk patients, it is necessary to identify whether continued sexual activity may pose a significant risk. If this is the case, the EAU recommends stopping sexual activity until cardiac status has been stabilised by treatment. GPs may also use validated questionnaires like the Sexual Health Inventory for Men (SHIM) to aid the assessment of sexual function.

Is There Evidence That a Low-Cost Drug for Erectile Dysfunction Has Been Prescribed, Such as Generic Sildenafil or Tadalafil Tablets? (90%)

Depending on the complexity and underlying cause of ED, patients have multiple choices of treatment ranging from intracavernosal injections to vacuum erection devices. However, oral PDE5Is are now recommended as the first-line medication for ED because of continuous advancements in safety, efficacy, and non-invasiveness. These drugs function by relaxing smooth muscle in the corpora cavernosa, which causes increased vascular filling of the penile blood vessels.

Examples of PDE5Is that are commonly used in primary care include sildenafil, tadalafil, vardenafil, and avanafil. While these medications do not share the same pharmacokinetic properties, evidence is not yet conclusive to suggest significant differences in efficacy and tolerability. Although two meta-analyses demonstrated that sildenafil and tadalafil may result in greater figures of patient satisfaction due to flexibility and ease of access [7,8]. In regard to cost, data from OpenPrescribing has classified sildenafil and tadalafil as being considerably cheaper than other PDE5Is, and advises that these should preferably be offered as first-line agents [4].

## Results

A total of 50 patients were included in this audit. Overall performance against national guidelines (BSSM & EUA [1,7]) varied across the three assessed criteria, with one standard achieved and two falling slightly below target thresholds.

Regarding investigation of underlying causes, 41 out of 50 patients (n = 41, 82%) underwent appropriate biochemical testing, including serum haemoglobin A1c (HbA1c), lipid profile, and testosterone levels. Although this represents the majority of the cohort, it did not meet the predefined audit standard of 85%. Review of patient records indicated that incomplete testing was often due to partial investigation panels rather than a complete absence of blood tests. These findings are summarised in Table 1.

**Table 1:** Assessment and management of ED at Elmwood Family Doctors according to national guidelines. CVD: cardiovascular disease; ED: erectile dysfunction.

Criteria	Total No. of patients	No. of patients achieved criteria	Audit performance (%)	Audit standard (%)
Arranged blood tests	50	41	82	85
CVD risk assessment	50	38	76	80
Low-cost prescribing	50	45	90	90

**Figure 1:**
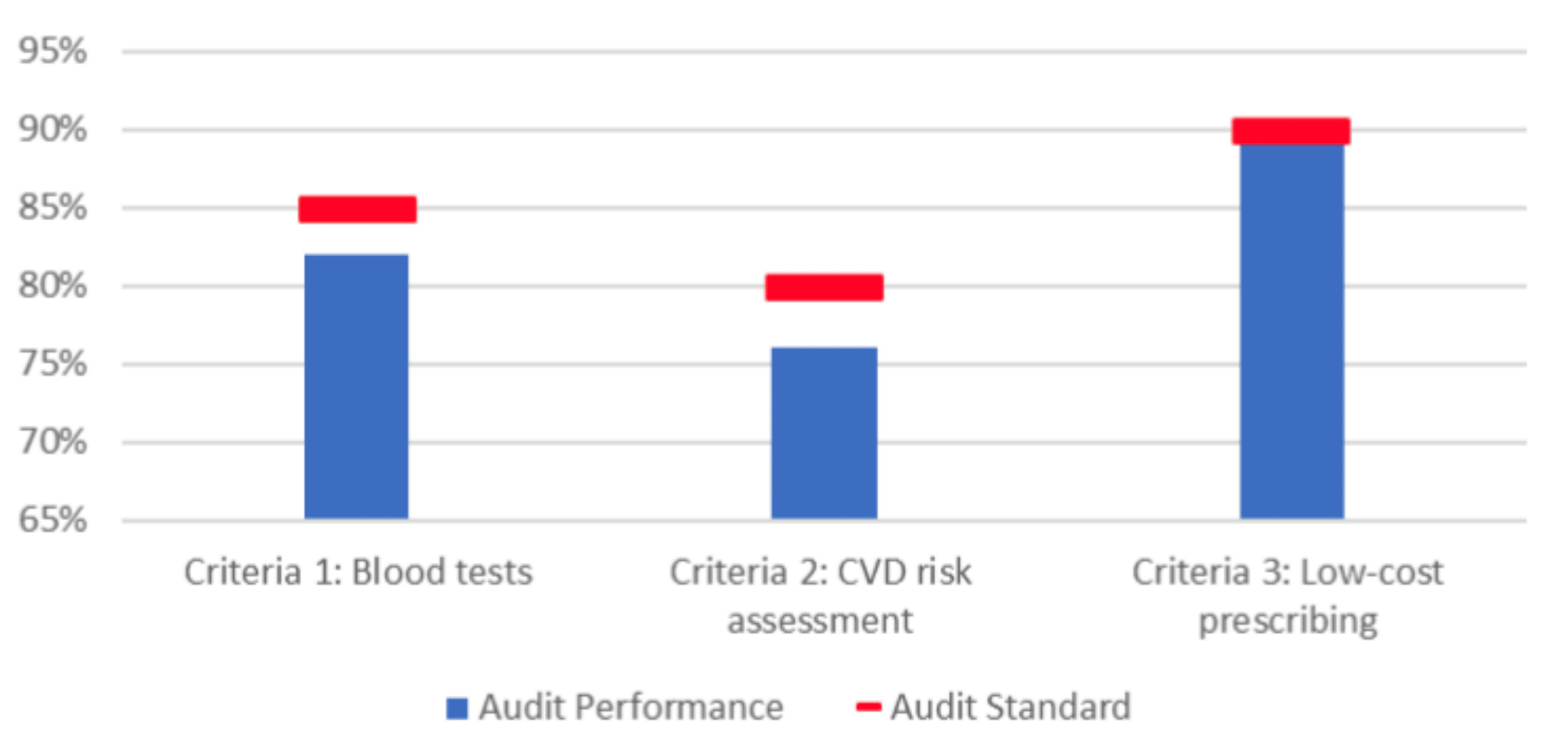
Comparison between audit performance and set standards for ED assessment and management. CVD: cardiovascular disease; ED: erectile dysfunction.

Cardiovascular risk assessment was documented in 38 out of 50 patients (n = 38, 76%), which also fell short of the target standard of 80%. Notably, there was an interesting pattern observed between patients who did not receive full biochemical investigations and those who lacked documented cardiovascular assessment, suggesting a potential gap in holistic evaluation during consultations. A comparison between achieved audit performance and predefined standards across all criteria is illustrated in Figure 1.

In contrast, prescribing practices were largely compliant with current recommendations. A total of 45 out of 50 patients (n = 45, 90%) were prescribed low-cost first-line PDE5Is, such as generic sildenafil or tadalafil, thereby meeting the audit standard of 90%. The remaining patients were prescribed alternative therapies, which may reflect individual clinical considerations or contraindications.

Overall, these results demonstrate good adherence to prescribing guidance but highlight areas for improvement in comprehensive investigation and cardiovascular risk assessment. The distribution of outcomes across all three criteria is presented in Table 1, while Figure 1 provides a visual comparison between observed performance and expected standards.

## Discussion

The results of this audit indicate that the quality of ED assessment and management at Elmwood Family Doctors is positive, although there is scope for improvement as the standards for two criteria were not fulfilled. A full blood screen was not performed on nine patients in the sample; further examination of records revealed that they received at least one of the tests, but were missing the complete panel of serum HbA1c, serum lipid profile, and total serum testosterone. Two of the patients in this category were also documented to have declined blood tests, despite being informed about the potential risk of underlying causes.

CVD risk assessment was successfully completed in 76% of patients, compared to the set standard of 80%. An interesting finding was the overlap between the group that did not have blood tests and those with no CVD evaluation. Analysis of individual records illustrated that the majority of patients either met both criteria or none. This pattern suggests that a comprehensive cardiovascular review may prompt appropriate biochemical investigation, or conversely, that a superficial consultation results in neither. From a patient perspective, an explanation of the link between CVD and ED as part of the consultation would increase their awareness and likelihood of compliance with investigations.

The standard for low-cost prescribing was achieved, with 90% of patients on generic sildenafil or tadalafil. The remainder of patients in this sample were on alternative treatments such as alprostadil injections and vardenafil. In certain cases, the use of such therapies may be appropriate as PDE5Is have reduced efficacy for ED that is severely vasculogenic or caused by post-radical prostatectomy. Discussions about ED management should occur more frequently during medication reviews to guide the selection of the most suitable treatment for the patient. For example, PDE5Is should not be prescribed in men who use nitrates as required for angina due to the increased risk of hypotensive events [9]. Hence, the maintenance of low-cost prescribing in these patients is challenging.

In retrospect, future audits may concentrate on using specific scoring tools such as SHIM or the Framingham Risk Score (FRS). Occasionally, brief and informal conversations regarding the patient’s medical history occur without documentation, which generates difficulties in obtaining reliable audit findings. The criteria for this audit studied “evidence of cardiovascular risk assessment” as a general overview, and narrowing the focus with the aforementioned tools could provide more accurate results.

Other examples of criteria can incorporate the role of lifestyle changes, such as weight loss or smoking, since these tend to precede or accompany pharmacological ED management [10]. In fact, a study in the United States discovered that only 25.4% of men diagnosed with ED were prescribed medications [11]. This signifies that, for future audits, the target population should be broadened to consist of patients managing their ED via risk factor modification and psychosexual therapies. The interactions between conservative and pharmacological treatments may offer promising insights for shared decision-making with the patient.

This project has several limitations. The routine measurement of testosterone is controversial, and studies have illustrated that the prevalence of lower levels in men with ED varies widely [9]. BSSM guidelines also recommend that testosterone blood tests be carried out between 9:00 and 11:00 am, and in this audit, information was not recorded on the timing of samples [1]. This audit was also limited by its single-centre design, as data were collected from one general practice only, which may reduce the representativeness of findings across other primary care settings with different patient demographics and prescribing patterns. Additionally, no formal statistical testing was performed because the project was designed as a descriptive quality improvement audit; therefore, the significance of observed patterns between variables could not be formally assessed.

Another important limitation of this audit is the selection bias introduced by only including patients who had been prescribed ED medication within the previous 12 months. This excludes patients managed conservatively through lifestyle modification, psychosexual therapy, or watchful waiting, and may have artificially inflated the findings related to prescribing practices while limiting the representativeness of the results for the wider population of patients presenting with ED in primary care. Lastly, while low-cost prescribing is optimal, direct comparative studies on the effectiveness of PDE5Is are lacking, and the opportunity to try different medications should still be encouraged according to patient circumstances [12].

Recommendations

The results of this audit were presented to the practice team during a weekly clinical meeting, and several changes were proposed to improve adherence to national guidelines. The use of a standardised diagnostic tool such as the International Index of Erectile Function (IIEF-5) during consultations was recommended to better classify severity and guide management decisions.

It was also advised that patients presenting with ED should be formally stratified into low-, intermediate-, or high-risk cardiovascular categories, in accordance with the Princeton III Consensus [13]. This would involve a structured assessment of angina severity, degree of heart failure using the New York Heart Association (NYHA) classification, blood pressure control, and the presence of high-risk arrhythmias.

In addition, modifications to the existing electronic ED template were suggested, including the incorporation of specific coding for blood tests to improve documentation and audit reliability. Patients should also be counselled regarding the availability of over-the-counter sildenafil and the risks associated with counterfeit medications from unregulated sources.

Finally, a medication review strategy was proposed to identify patients prescribed branded treatments such as Cialis or Viagra, with a view to switching to a generic drug after effective patient counselling and consensus, which can aid in improving medication adherence and reducing healthcare costs.

A re-audit should be conducted within 12 months to assess the impact of these interventions. Once again, this re-audit must be for those patients who meet the selected criteria of being medicated and first diagnosis of ED no earlier than 2017. Furthermore, the practice can follow up on those who received their ED diagnosis before 2017, and implement these recommendations so that their management can be up to date with the most recent NICE guidelines.

Sustainability impact

Quality improvement efforts must acknowledge the value of patient outcomes against a "triple bottom line" of social, financial, and environmental impacts. By assessing care using holistic principles such as disease prevention, patient empowerment, and lean pathways, audits can promote the growth of ethical health services in a community setting. In the case of ED, structured reviews using well-designed templates could help establish patient adherence to medication and whether lifestyle interventions have made a substantial improvement.

The National Health Service (NHS) has committed to satisfy carbon reduction targets, and a major component of these projects is sustainable prescribing. Reviews have approximated that at least 10% of prescribed items in UK primary care were unnecessary [14]. By incentivising weight loss, alcohol reduction, and smoking cessation, patients may be able to conservatively treat their ED, and these measures should be suggested as first-line for environmental benefit. From a financial viewpoint, this audit advocated for the use of inexpensive and generic sildenafil and tadalafil. GPs should notify patients about these medications being cheaper to acquire in relation to their branded counterparts.

The social dimensions revolve around education and reflection to ensure that patients are well-informed about their ED treatment. The integration of cardiovascular health issues into ED consultations may further motivate patients to commence changes like smoking cessation, which has been shown to decrease levels of social connections and overall well-being [15].

## Conclusions

This audit demonstrated that while the management of ED in this general practice is broadly aligned with national guidelines, there are notable gaps in the assessment of underlying causes and cardiovascular risk. Although prescribing practices met expected standards, improvements are required in the consistent use of blood investigations and structured cardiovascular evaluation. It is also important to note that, as a single-centre audit with a unique demographic (higher proportion of elderly and chronically ill patients), the findings at Elmwood Family Doctors may not be generalisable to other primary care settings.

Targeted interventions, including the use of standardised assessment tools, enhanced documentation templates, and clinician education, may improve compliance with recommended practice. A re-audit following implementation of these measures will be essential to determine sustained improvement in patient care.
